# Physicochemical Characterization of Nanofillers for Unsaturated Polyester Resin Modification

**DOI:** 10.3390/ma19173720

**Published:** 2026-08-31

**Authors:** Dominik Stępka, Karina Niziołek, Dagmara Słota, Josef Jampilek, Agnieszka Sobczak-Kupiec

**Affiliations:** 1 Cracow University of Technology, CUT Doctoral School, Faculty of Materials Engineering and Physics, Department of Materials Science, 37 Jana Pawła II Av., 31-864 Krakow, Poland; dominik.stepka@doktorant.pk.edu.pl (D.S.); karina.niziolek@pk.edu.pl (K.N.); 2 Cracow University of Technology, Faculty of Materials Engineering and Physics, Department of Materials Science, 37 Jana Pawła II Av., 31-864 Krakow, Poland; agnieszka.sobczak-kupiec@pk.edu.pl; 3 Department of Analytical Chemistry, Faculty of Natural Sciences, Comenius University, Ilkovicova 6, 842 15 Bratislava, Slovakia; 4 Department of Chemical Biology, Faculty of Science, Palacky University, Slechtitelu 27, 779 00 Olomouc, Czech Republic

**Keywords:** nanofillers, carbon nanotubes, nanoclay, halloysite, cloisite, zinc oxide, unsaturated polyester resins

## Abstract

Nanofillers are widely used to enhance the performance of polymer composites; however, their effectiveness strongly depends on their physicochemical properties and dispersion behavior. In this study, five commercially available nanofillers, namely multi-walled carbon nanotubes (CNTs), nanoclay, halloysite, Cloisite^®^ 30B, and zinc oxide (ZnO), were comprehensively characterized as potential modifiers of unsaturated polyester resin. The results confirm the characteristic chemical composition and crystalline structure of all investigated nanomaterials. CNTs exhibited the highest specific surface area (213.7 m^2^ g^−1^) and pore volume, whereas ZnO showed the lowest median equivalent particle diameter determined by laser diffraction (D_50_ = 2.09 μm). The clay-based fillers displayed comparable particle size distributions, with D_50_ values ranging from 8.29 to 9.00 μm. SEM observations revealed substantial differences in morphology and agglomeration state, with CNTs forming compact entangled agglomerates, while ZnO exhibited the most homogeneous microstructure. Suspension stability analysis demonstrated that particle size alone could not explain the observed sedimentation behavior. ZnO exhibited the highest dispersion stability, whereas halloysite and Cloisite showed progressive sedimentation. Nanoclay displayed delayed destabilization associated with agglomeration processes, while CNT suspensions were governed by structural rearrangements rather than classical sedimentation. The obtained results indicate that the dispersion behavior of nanofillers in unsaturated polyester resin is associated with differences in particle size, morphology, and agglomeration tendency. The presented comparative characterization provides a basis for the rational selection of nanofillers for polyester resin-based nanocomposites.

## 1. Introduction

Unsaturated polyester resins (UPRs) are among the most widely used thermosetting polymers due to their favorable balance of mechanical properties, chemical resistance, low cost, and ease of processing [1,2]. These materials are extensively applied in construction, transportation, marine, and electrical industries, where lightweight structures with good mechanical performance are required [3,4,5]. Despite their numerous advantages, UPR-based materials exhibit certain limitations, including relatively low fracture toughness, limited thermal stability, and susceptibility to crack propagation under mechanical loading [6,7,8,9,10]. One of the most effective approaches for improving the properties of polymer matrices involves the incorporation of nanofillers [11,12]. Owing to their high specific surface area and large surface-to-volume ratio, nanomaterials can significantly influence the microstructure and interfacial interactions within polymer composites, even at very low loading levels [13,14]. Numerous studies have demonstrated that the addition of nanofillers may improve the mechanical, thermal, barrier, tribological, and functional properties of polymer materials, enabling the development of polymer nanocomposites with enhanced performance [15,16,17]. However, the extent of these improvements strongly depends on the physicochemical characteristics of the applied nanofillers, including their particle size distribution, morphology, crystallographic structure, surface area, pore characteristics, and tendency to form agglomerates [18,19].

Among the various nanofillers investigated for polymer modification, carbon nanotubes (CNTs), layered aluminosilicates, and metal oxide nanoparticles are among the most promising materials. Carbon nanotubes are characterized by exceptional tensile strength, high aspect ratio, and remarkable electrical and thermal conductivity, making them attractive reinforcing agents for advanced polymer composites [20,21,22]. However, their effective utilization is often limited by their tendency to form agglomerates, which may hinder uniform dispersion within the polymer matrix [23,24]. Layered aluminosilicates, including nanoclay, Cloisite, and halloysite, have attracted considerable interest due to their ability to improve the mechanical, thermal, and barrier properties of polymeric materials [25]. Their effectiveness is primarily associated with their layered structure, high surface area, and potential to interact with polymer chains at the nanoscale [26,27,28]. Halloysite is particularly interesting because of its naturally occurring tubular morphology, which distinguishes it from conventional layered clays and offers additional possibilities for filler—matrix interactions [29,30,31]. In contrast, Cloisite and nanoclay are commonly used to enhance stiffness, dimensional stability, and resistance to gas and moisture permeation [32,33]. Metal oxide nanoparticles, such as zinc oxide (ZnO), constitute another important group of nanofillers used in polymer engineering. ZnO nanoparticles have been extensively studied due to their high thermal stability, ultraviolet shielding capability, antimicrobial activity, and potential to improve the durability of polymeric materials [34,35]. As a result, ZnO has found applications in multifunctional polymer nanocomposites intended for structural, protective, and biomedical applications [36,37,38].

Although numerous studies have investigated the influence of individual nanofillers on the properties of polymer composites, comparative analyses of different classes of nanomaterials performed under identical experimental conditions remain relatively limited. Most available reports focus primarily on the performance of the final composite materials, whereas considerably less attention has been devoted to a systematic comparison of the structural, surface, and morphological characteristics of the nanofillers themselves. Since the effectiveness of a nanofiller strongly depends on its crystallinity, particle size distribution, surface area, pore structure, morphology, and dispersion behavior, comprehensive characterization is essential for understanding its potential performance in polymer matrices.

Therefore, the aim of this study was to perform a comparative physicochemical characterization of commercially available CNTs, nanoclay, halloysite, Cloisite, and ZnO, which are widely used as reinforcing nanofillers for the fabrication of polymer nanocomposites. The investigated materials were comprehensively analyzed using Fourier transform infrared spectroscopy (FTIR), X-ray diffraction (XRD), nitrogen adsorption–desorption analysis (BET), scanning electron microscopy coupled with energy-dispersive X-ray spectroscopy (SEM-EDS), particle size distribution analysis, and suspension stability measurements in an unsaturated polyester resin matrix. Particular attention was devoted to comparing the structural, morphological, and textural characteristics of the investigated nanofillers in relation to their dispersion behavior in the resin. The obtained results provide a scientific basis for selecting nanofillers with suitable physicochemical characteristics and dispersion behavior prior to composite fabrication.

The novelty of this study lies in the systematic comparison of five commercially relevant nanofillers with markedly different chemical compositions, morphologies, and dimensional characteristics under identical experimental and resin-processing conditions. Rather than evaluating each nanofiller individually, the applied comparative approach enables direct assessment of how these material-specific characteristics are reflected in their dispersion and stability behavior within the same unsaturated polyester resin. This provides a consistent experimental basis for the preliminary selection of nanofillers for further development of polyester-based composites, including materials intended for composite pipe applications.

## 2. Materials and Methods

### 2.1. Materials

The nanofillers used in this study included nanoclay, halloysite, Cloisite^®^ 30B, multi-walled carbon nanotubes (CNTs), and zinc oxide (ZnO). Nanoclay (montmorillonite clay, CAS No. 1318-93-0) and halloysite nanoclay (CAS No. 1332-58-7) were purchased from Sigma-Aldrich (St. Louis, MO, USA). According to the manufacturer, the nanoclay consisted of montmorillonite modified with 34–45 wt.% dimethyl dialkyl amine. The average particle size was below 20 μm and the bulk density ranged from 200 to 500 kg/m^3^. Cloisite^®^ 30B, an organically modified montmorillonite, was used as received. Multi-walled carbon nanotubes (MWCNTs, CAS No. 308068-56-6, product no. 412988, Sigma-Aldrich, St. Louis, MO, USA) were employed as the carbon-based nanofiller. According to the manufacturer, the material was produced by the arc-discharge method and supplied as an as-produced cathode deposit with a nominal outer diameter of 7–15 nm, a length of 0.5–10 μm, and an average number of 5–20 graphitic layers. The material contained >99% total carbon (TGA) and >7.5% MWCNTs and was specified by the manufacturer as catalyst-free. No surface functionalization was specified by the manufacturer. Zinc oxide (ZnO, CAS No. 1314-13-2) was supplied by Sigma-Aldrich (St. Louis, MO, USA). The polymer matrix was an orthophthalic unsaturated polyester resin (UPR) supplied by Ciech Sarzyna S.A. (Nowa Sarzyna, Poland) under the trade name POLIMAL^®^ 109A. The resin was used as received without further purification.

### 2.2. Fourier Transform Infrared Spectroscopy Analysis

Fourier transform infrared spectroscopy (FT-IR) was employed to identify the characteristic functional groups and chemical bonds present in the investigated nanomaterials. FT-IR spectra were recorded using a Nicolet iS5 FTIR spectrometer equipped with an iD7 ATR accessory (Thermo Scientific, Loughborough, UK). Measurements were performed in the spectral range of 4000–400 cm^−1^ with a resolution of 4 cm^−1^ and 32 accumulated scans at room temperature.

### 2.3. X-Ray Diffraction Analysis

The phase composition and crystalline structure of the investigated nanomaterials were analyzed by X-ray diffraction (XRD). The diffraction patterns were used to identify crystalline phases and evaluate the structural characteristics of the powders. Measurements were performed using an Aeris X-ray diffractometer equipped with a PIXcel1D-Medipix3 detector (Malvern Panalytical, Malvern, UK) and Cu Kα radiation (λ = 1.5406 Å). Data were collected over a 2θ range of 10–100° with a step size of 0.0027° and a counting time of 340.425 s per step, ensuring high-quality diffraction data suitable for detailed phase analysis.

### 2.4. Specific Surface Area and Porosity Studies

The textural properties of the investigated nanomaterials were determined by low-temperature nitrogen adsorption–desorption measurements at 77.35 K using an Autosorb iQ analyzer (Anton Paar, Graz, Austria). Prior to the measurements, the samples were degassed under vacuum using a multistep heating procedure: at 80 °C (2 °C min^−1^, 30 min), followed by 120 °C (2 °C min^−1^, 30 min), and finally at 300 °C (5 °C min^−1^, 400 min). The same degassing protocol was applied to all materials to maintain consistent measurement conditions. For organomodified clay samples, the possibility of thermally induced changes in the organic modifier during the final degassing step cannot be excluded.

Nitrogen adsorption–desorption isotherms were recorded over a relative pressure (p/p_0_) range from 1 × 10^−7^ to 0.99. The specific surface area (S_BET_) was calculated using the Brunauer–Emmett–Teller (BET) method within the relative pressure range of 0.005–0.05. Micropore characteristics, including the micropore surface area (S_micro_) and micropore volume (V_micro_), were determined using the t-plot method with the de Boer statistical thickness model. The external specific surface area (S_ext_), corresponding to the mesoporous and external particle surface, was also obtained from the t-plot analysis.

The pore size distribution was evaluated using the non-local density functional theory (NLDFT) Equilibrium model. For CNTs, the pore-size distribution was calculated using an NLDFT equilibrium kernel for N_2_ adsorption at 77 K on carbon, assuming slit/cylindrical pore geometry. Based on this model, the cumulative pore surface area (S_cum_), cumulative pore volume (V_cum_), and the modal pore diameter (D_mode_) were determined. All calculations were performed using Kaomi software (version 2.0, An-ton Paar, Graz, Austria).

### 2.5. Particle Size Analysis

The particle size distribution of the investigated nanomaterials was determined using a PSA 1190 LD laser diffraction particle size analyzer (Anton Paar GmbH, Graz, Austria). Distilled water was used as the dispersing medium. Measurements were performed within the particle size range of 0.04–2500 μm. For each material, three independent measurements were conducted, and the average particle size distribution was calculated using Kalliope Professional software (version 2.22.1, Anton Paar GmbH, Graz, Austria). The characteristic particle diameters corresponding to 10%, 50%, and 90% of the cumulative particle size distribution (D10, D50, and D90) were determined and used for comparative analysis of the investigated materials. The obtained results were presented as particle size distribution curves and used to evaluate the particle size characteristics of the analyzed nanomaterials. For highly anisotropic materials such as CNTs, the values obtained by laser diffraction were interpreted as equivalent spherical diameters of dispersed bundles and agglomerates rather than as the actual nanotube diameters or lengths.

### 2.6. Morphology Analysis

The morphology and surface characteristics of the investigated nanomaterials were examined using scanning electron microscopy (SEM). In addition, elemental composition and elemental distribution were analyzed by energy-dispersive X-ray spectroscopy (EDS) and elemental mapping. The analyses were performed using a JEOL IT200 scanning electron microscope equipped with an EDS detector (JEOL Ltd., Tokyo, Japan). For SEM/EDS analysis, the powder samples were mounted on carbon adhesive tape. Prior to imaging, the powder samples were mounted on carbon adhesive tabs and sputter-coated with a thin gold layer using a DII-29030SCTR Smart Coater (JEOL Ltd., Tokyo, Japan) to improve surface conductivity. The obtained micrographs were used to evaluate particle morphology, agglomeration behavior, and structural features of the investigated nanomaterials, while EDS analysis provided qualitative information on their elemental composition.

### 2.7. Synthesis and Modification of Resin Samples

For sample preparation, 30 g of orthophthalic unsaturated polyester resin was placed in a glass vessel and mixed with an appropriate amount of nanopowder corresponding to 0.1 wt.%. A fixed nanofiller concentration of 0.1 wt.% was used for all systems to provide a common mass-based reference and enable direct comparison of their dispersion behavior under identical preparation and measurement conditions. The mixture was thoroughly stirred, and the vessel was tightly sealed. All procedures were carried out in a fume hood with appropriate safety precautions. Subsequently, the sealed vessels containing 30 g of the resin/nanofiller suspensions were subjected to ultrasonic treatment using a PROCLEAN 10.0S ultrasonic bath (Ulsonix Cleaning Instruments, Zielona Góra, Poland). Each suspension was placed in a sealed cylindrical glass vessel with a nominal capacity of 30 mL (75 mm in height and 27 mm in diameter), positioned centrally in the ultrasonic bath using the basket. Sonication was performed for 10 min at a frequency of 40 kHz and a nominal ultrasonic power of 240 W. The bath had a capacity of 10 L and internal tank dimensions of 150 × 240 × 300 mm. The bath temperature during sonication was approximately 23 °C. The reported ultrasonic power corresponds to the nominal power of the device.

### 2.8. Stability of Nano-Additives in Resin

Considering the intended application of the nanoparticles as modifiers of unsaturated polyester resin, the samples prepared according to Section 2.7 were subjected to stability analysis to evaluate the sedimentation behavior of the nanofillers in the resin matrix and to determine their dispersion stability over time. The measurements were carried out using a MultiScan MS20 analyzer (DataPhysics Instruments, Filderstad, Germany). For each nanofiller, 3 independently prepared suspensions were analyzed (*n* = 3) under identical experimental conditions to assess the reproducibility of the observed stability behavior. The analysis was performed over a period of 20 h, during which 10,000 scans were recorded at 7 s intervals. No curing agent was added to the resin during the suspension stability measurements; therefore, the investigated systems remained uncured throughout the 20 h test. Both transmission and backscattering signals were monitored to assess the sedimentation processes of the nanoparticles within the resin.

## 3. Results

### 3.1. Fourier Transform Infrared Spectroscopy Analysis

**Figure 1:**
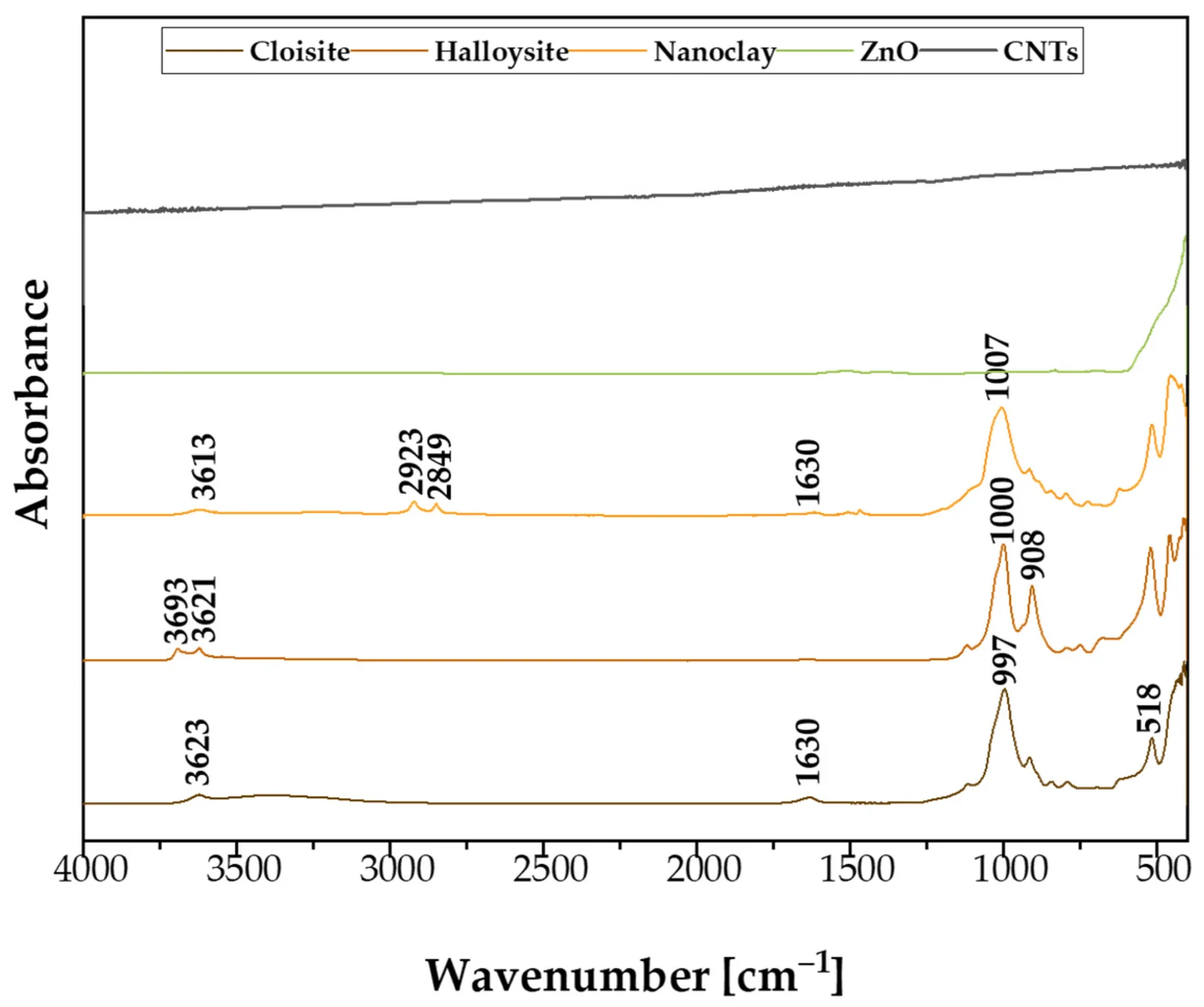
FTIR spectra of the investigated nanopowders.

The FTIR spectra of the four nanopowders are presented in Figure 1. For cloisite, the broad band in the 3600–3000 cm^−1^ region can be assigned to O-H stretching vibrations of structural hydroxyl groups and physically adsorbed water. The band at 3623 cm^−1^ is associated with Si-OH. The band near 1630 cm^−1^ corresponds to H-O-H bending vibrations of adsorbed water. The intense absorption at 997 cm^−1^ is attributed to Si-O-Si stretching vibrations, while bands 518 cm^−1^ are related to the stretching modes of Al-O [39,40]. The halloysite spectrum exhibits distinct bands near 3693 and 3621 cm^−1^, assigned inner hydroxyl groups. The strong absorption band around 1000 cm^−1^ corresponds to Si-O stretching, whereas the bands near 908 cm^−1^ and below 793 cm^−1^ are associated with Al-OH bending and Si-O-Al and Al-O vibrations, respectively. Peak at 518 cm^−1^ is attributed to Al-O-Si vibration. These features confirm the typical aluminosilicate structure of halloysite [41,42].

In the nanoclay spectrum, similarly to cloisite and halloysite, the band in the hydroxyl region indicates the presence of surface -OH groups and adsorbed moisture at 3613 cm^−1^. The absorption around 1630 cm^−1^ is assigned to bending vibrations of molecular water. Singals at 2923 cm^−1^ and 2849 cm^−1^ were due to asymmetric and symmetric vibration of methylene group (CH_2_) in the organic compound. The intense band at 1007 cm^−1^ originates from Si-O stretching vibrations of the silicate framework, while bands in the lower wavenumber region correspond to Si-O-Al and metal-oxygen lattice vibrations [43,44]. The ZnO spectrum differs significantly from the clay-based materials. The strong absorptions below 593 cm^−1^ is characteristic of Zn-O stretching vibrations, confirming the formation of zinc oxide. The absence of intense silicate bands around 1000 cm^−1^ further distinguishes ZnO from other tested nanopowders [45].

Overall, the FTIR results confirm the successful identification of the studied nanopowders. Cloisite, halloysite and nanoclay exhibit characteristic bands of layered aluminosilicates, including O-H, H-O-H, Si-O-Si, Al-OH and Si-O-Al vibrations, whereas ZnO is identified by the dominant Zn-O lattice vibration in the low-wavenumber region. In contrast to the other investigated nanofillers, the FTIR spectrum of CNTs exhibited very low spectral intensity and no clearly resolved characteristic absorption bands over the investigated wavenumber range. In particular, no distinct bands that could be unambiguously assigned to oxygen-containing surface functional groups were identified. Therefore, the FTIR results do not provide direct evidence of pronounced surface functionalization of the investigated CNTs.

### 3.2. X-Ray Diffraction Analysis

**Figure 2:**
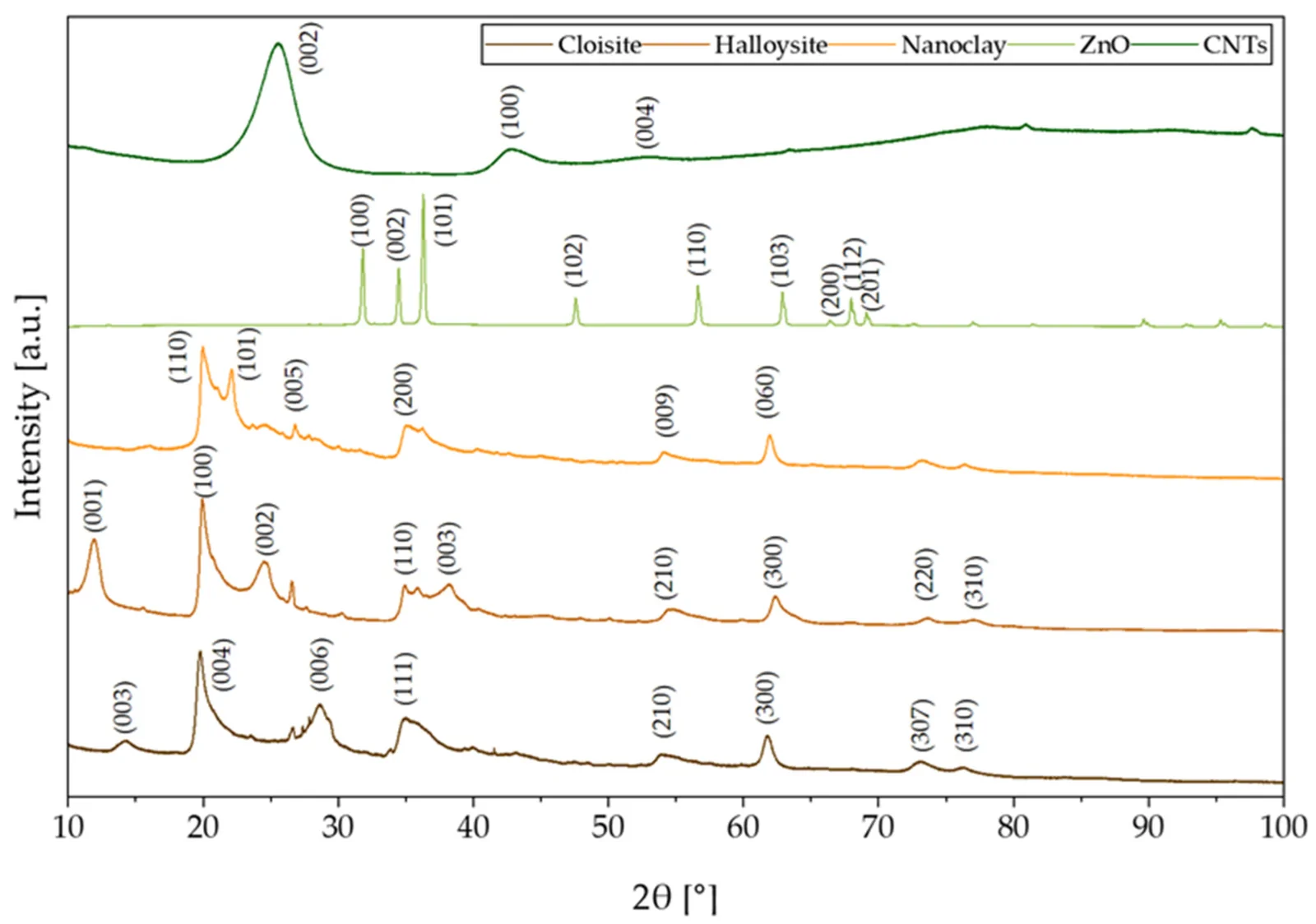
Diffractograms of nanopowders.

The X-ray diffraction patterns of Cloisite, halloysite, nanoclay, ZnO, and carbon nanotubes (CNTs) are presented in Figure 2. The obtained diffraction profiles were compared with the reference patterns available in the International Centre for Diffraction Data (ICDD) database. The experimental diffraction peaks showed good agreement with the reference cards corresponding to Cloisite (ICDD No. 00-012-0219), halloysite (ICDD No. 00-009-0451), nanoclay (ICDD No. 00-075-0577), ZnO (ICDD No. 04-008-7114), and CNTs (ICDD No. 00-058-1638), confirming the phase composition and crystalline structure of the investigated nanomaterials.

The diffraction pattern of Cloisite exhibited characteristic reflections at approximately 19.8°, 26.8°, 35.4°, 54.3°, and 62.1° 2θ, corresponding to the crystallographic planes (004), (006), (111), (210), and (300), respectively [46,47]. Additional reflections observed at higher diffraction angles further confirmed the crystalline layered aluminosilicate structure. Halloysite displayed characteristic reflections at approximately 11.8°, 20.0°, and 24.8° 2θ, assigned to the (001), (100), and (002) planes, respectively [48,49]. The remaining diffraction peaks were consistent with the reference halloysite pattern, indicating the presence of a well-defined layered aluminosilicate structure. The nanoclay sample exhibited characteristic reflections associated with layered silicates, with diffraction peaks observed at approximately 18.5°, 20.5°, 25.5°, 35.0°, 54.0°, and 62.0° 2θ [50,51]. The good agreement with the ICDD reference pattern confirmed the crystalline nature of the material. The ZnO sample showed a series of sharp and intense diffraction peaks located at approximately 31.8°, 34.4°, 36.3°, 47.5°, 56.6°, 62.8°, 66.4°, 67.9°, and 69.1° 2θ, corresponding to the (100), (002), (101), (102), (110), (103), (200), (112), and (201) crystallographic planes, respectively [52,53]. These reflections are characteristic of the hexagonal wurtzite crystal structure and are in excellent agreement with the ICDD reference card No. 04-008-7114. The diffraction pattern of CNTs was dominated by a strong reflection centered at approximately 26° 2θ, corresponding to the graphitic (002) plane [54]. Additional low-intensity reflections were observed near 43° and 54° 2θ, which can be attributed to the (100) and (004) planes of graphitic carbon [55]. The obtained pattern was consistent with the reference CNTs diffraction data (ICDD No. 00-058-1638).

Overall, the XRD analysis confirmed the characteristic crystalline structures of all investigated nanomaterials and demonstrated good agreement between the experimental diffraction patterns and the corresponding ICDD reference data.

### 3.3. Specific Surface Area and Porosity Studies

The specific surface area and pore structure parameters of the investigated nanomaterials are summarized in Table 1. All materials exhibited distinct adsorption–desorption behaviors, indicating differences in their surface characteristics and pore structures.

Among the investigated nanomaterials, CNTs exhibited the highest specific surface area (S_BET_ = 213.728 m^2^/g), followed by Cloisite (63.671 m^2^/g), halloysite (51.828 m^2^/g), nanoclay (23.345 m^2^/g), and ZnO (10.547 m^2^/g). A similar trend was observed for the cumulative surface area (S_cum_), with values of 195.373, 88.508, 48.349, 22.356, and 10.114 m^2^/g for CNTs, Cloisite, halloysite, nanoclay, and ZnO, respectively. The t-plot analysis revealed no detectable micropore surface area (S_micro_) or micropore volume (V_micro_) for halloysite, nanoclay, ZnO, and CNTs. In contrast, Cloisite exhibited a measurable microporous contribution, with S_micro_ and V_micro_ values of 25.886 m^2^/g and 0.012 cm^3^/g, respectively. Consequently, the external surface area (S_ext_) of Cloisite (38.039 m^2^/g) was lower than its total BET surface area, whereas for the remaining materials S_ext was identical to S_BET_. The cumulative pore volume (V_cum_) also differed among the investigated nanomaterials, reaching 0.452 cm^3^/g for CNTs, 0.110 cm^3^/g for halloysite, 0.054 cm^3^/g for Cloisite, 0.046 cm^3^/g for nanoclay, and 0.012 cm^3^/g for ZnO. Considerable differences were also observed in the modal pore diameter (D_mode_). Cloisite exhibited the largest dominant pore diameter (63.671 nm), followed by nanoclay (23.345 nm), halloysite (12.991 nm), ZnO (3.537 nm), and CNTs (0.823 nm). For CNTs, the NLDFT analysis yielded a modal pore width of 0.823 nm, whereas the t-plot analysis indicated no measurable micropore surface area or micropore volume.

**Figure 3:**
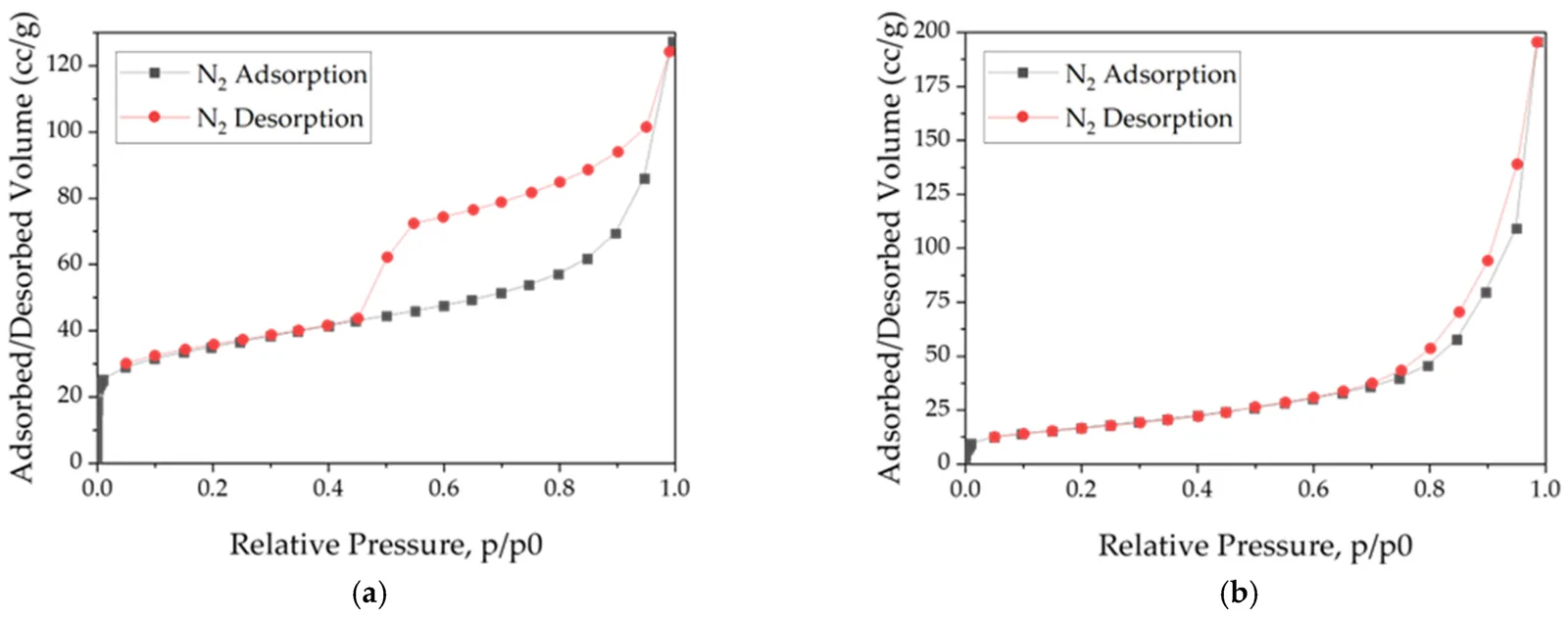

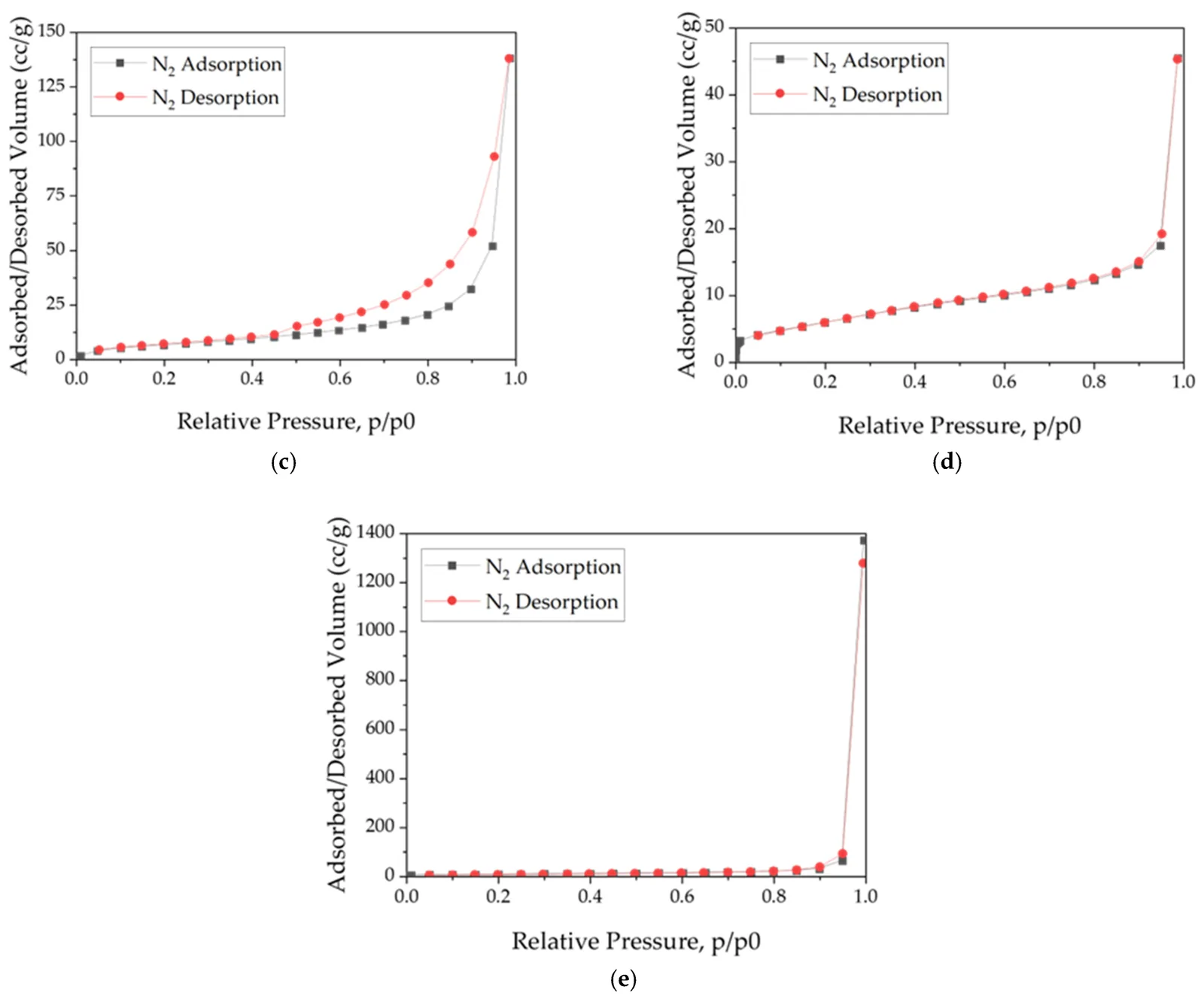
BET nitrogen adsorption–desorption isotherm plot: (**a**) cloisite; (**b**) halloysite; (**c**) nanoclay; (**d**) ZnO; and (**e**) CNTs.

The adsorption–desorption isotherms (Figure 3) further confirmed the differences in the textural characteristics of the analyzed nanomaterials. Cloisite exhibited the most pronounced hysteresis loop, while halloysite, nanoclay, and ZnO showed a gradual increase in nitrogen adsorption followed by a sharp uptake at high relative pressures. In contrast, CNTs displayed a relatively low adsorption over most of the investigated pressure range, followed by a rapid increase near the saturation pressure, resulting in the highest total adsorbed nitrogen volume among all investigated materials.

### 3.4. Particle Size Analysis

**Figure 4:**
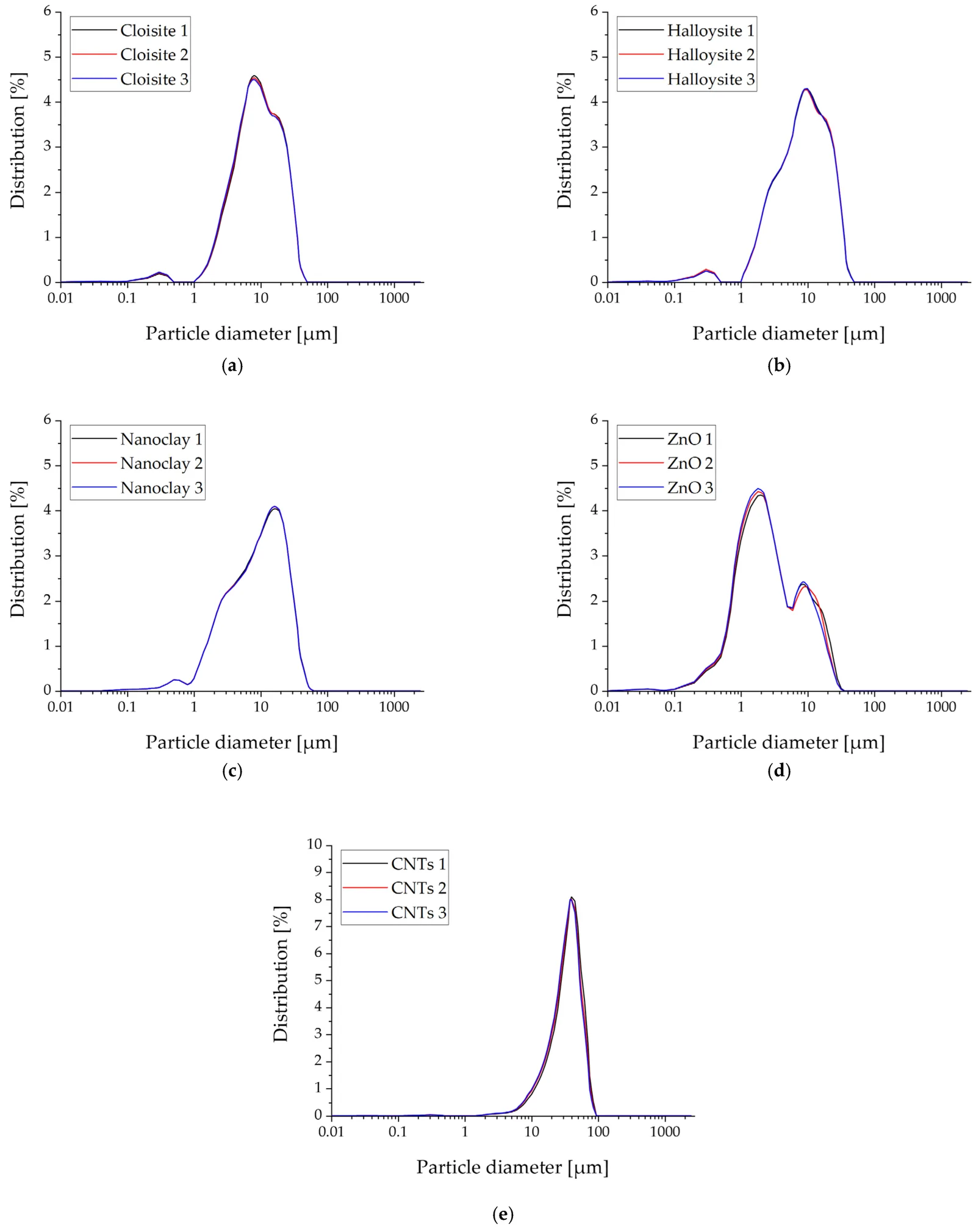
Histogram of particle size distribution for nanopowders: (**a**) cloisite; (**b**) halloysite; (**c**) nanoclay; (**d**) ZnO; and (**e**) CNTs.

The particle size distributions of the investigated nanofillers obtained by laser diffraction are presented in Figure 4, while the corresponding characteristic particle size parameters are summarized in Table 2. The three independent measurements performed for each material showed excellent reproducibility, as evidenced by the nearly overlapping distribution curves.

Cloisite exhibited a broad particle size distribution extending approximately from 1 to 100 μm, with the majority of particles concentrated within the 5–30 μm range (Figure 4a). The characteristic diameters were D_10_ = 2.825 ± 0.068 μm, D_50_ = 8.357 ± 0.106 μm, and D_90_ = 22.663 ± 0.063 μm, resulting in a mean particle size of 11.309 ± 0.083 μm. The distribution curve displayed a dominant peak centered at approximately 10–15 μm. A similar distribution profile was observed for halloysite (Figure 4b). The measured D_10_, D_50_, and D_90_ values were 2.227 ± 0.016 μm, 8.290 ± 0.041 μm, and 22.552 ± 0.091 μm, respectively, with a mean particle size of 11.033 ± 0.042 μm. The distribution maximum was located within the same particle size range as that observed for Cloisite, although a slightly higher contribution of particles below 1 μm was detected. Nanoclay exhibited the broadest particle size distribution among the clay-based fillers (Figure 4c). The corresponding D_10_, D_50_, and D_90_ values reached 1.940 ± 0.003 μm, 8.996 ± 0.034 μm, and 24.587 ± 0.073 μm, respectively, while the mean particle size was 11.988 ± 0.011 μm. Compared with Cloisite and halloysite, nanoclay showed slightly higher D_50_ and D_90_ values, indicating a shift of the distribution toward larger particle diameters.

The ZnO sample was characterized by the smallest particle dimensions among all investigated fillers. The D_10_, D_50_, and D_90_ values were 0.638 ± 0.019 μm, 2.086 ± 0.072 μm, and 11.475 ± 0.724 μm, respectively, with a mean particle size of 4.424 ± 0.229 μm. As shown in Figure 4d, the particle size distribution differed markedly from those of the clay-based materials and exhibited a bimodal profile with two distinct maxima located at approximately 2–3 μm and 8–10 μm.

The largest equivalent particle diameters determined by laser diffraction were recorded for CNTs (Figure 4e). The characteristic diameters reached D_10_ = 13.743 ± 0.707 μm, D_50_ = 33.511 ± 1.241 μm, and D_90_ = 55.582 ± 1.902 μm, while the mean particle size was 36.023 ± 1.287 μm. The distribution curve was characterized by a narrow and symmetrical peak centered at approximately 30–40 μm, indicating a relatively uniform range of equivalent agglomerate diameters under the applied measurement conditions.

A comparison of the median particle diameters revealed that the D_50_ value increased in the order: ZnO (2.086 μm) < Halloysite (8.290 μm) ≈ Cloisite (8.357 μm) < Nanoclay (8.996 μm) << CNTs (33.511 μm). Similarly, the mean equivalent particle size ranged from 4.424 μm for ZnO to 36.023 μm for CNTs, while the clay-based fillers exhibited comparable mean particle sizes of approximately 11 μm.

### 3.5. Morphology Analysis

**Figure 5:**
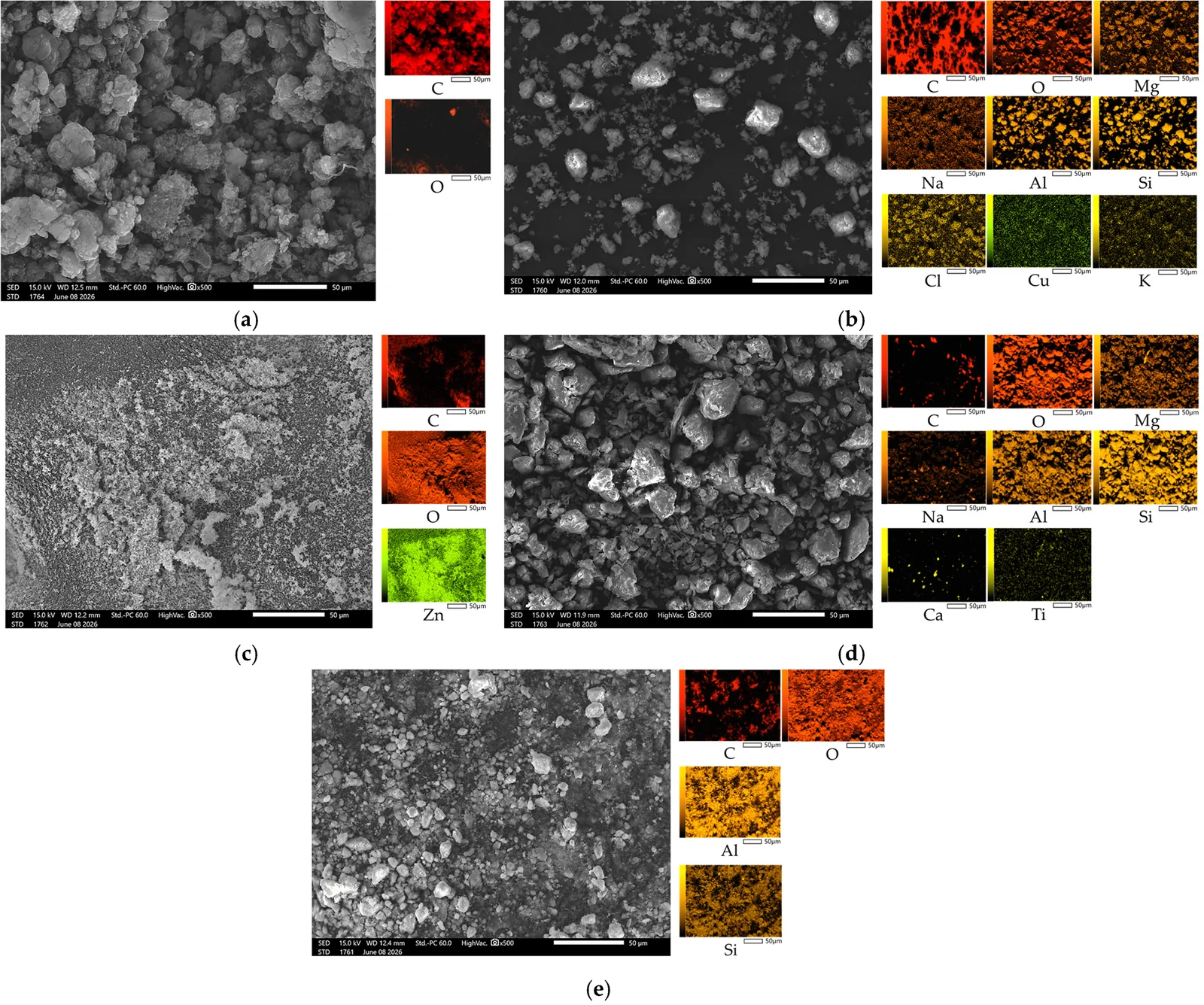
SEM morphology and corresponding elemental maps of the investigated nanopowders: (**a**) CNTs, (**b**) Nanoclay, (**c**) ZnO, (**d**) cloisite, and (**e**) halloysite.

The morphology and elemental composition of the investigated nanopowders were examined using SEM coupled elemental mapping (Figure 5).

SEM observations revealed differences in the morphology and agglomeration state of the analyzed materials. The ZnO sample exhibited the most homogeneous microstructure, characterized by relatively fine particles and small agglomerates distributed uniformly throughout the observed area. In contrast, cloisite and nanoclay displayed larger irregular aggregates typical of layered aluminosilicate materials. The nanoclay sample showed the broadest size distribution of agglomerates, with large aggregates surrounded by numerous smaller particles, indicating incomplete deagglomeration of the powder. Halloysite exhibited a more uniform particle distribution and a lower degree of agglomeration compared with cloisite. The CNTs sample was dominated by compact agglomerates and entangled structures, while individual nanotubes were not distinguishable due to the strong tendency of CNTs to form bundles through van der Waals interactions [56]. Elemental mapping confirmed the characteristic chemical composition of all investigated nanopowders. The CNT sample was composed predominantly of carbon with minor oxygen content, indicating the presence of oxygen-containing surface groups. The ZnO sample showed a homogeneous distribution of Zn and O throughout the analyzed area, confirming the chemical uniformity of the material. The aluminosilicate fillers were characterized by the presence of Si, Al, and O, accompanied by minor amounts of Mg, Na, K, Ca, Ti, and Cl depending on the material type. The relatively uniform distribution of these elements indicates the absence of significant compositional segregation within the analyzed powders.

Quantitative EDS analysis further confirmed the chemical composition of the investigated nanopowders (Table 3). CNTs were composed predominantly of carbon (92.36 wt.%), with a minor oxygen contribution (7.64 wt.%), which may be attributed to oxygen-containing surface functional groups and atmospheric oxidation. The ZnO sample was characterized by the presence of zinc (63.6 wt.%) and oxygen (17.9 wt.%), confirming the oxide nature of the material. The carbon signal detected in the ZnO sample should be interpreted with caution because the powder was mounted on carbon adhesive tape for SEM/EDS analysis. Thus, the measured carbon contribution may partially originate from the mounting substrate and affects the quantitative elemental fractions obtained by EDS. The aluminosilicate fillers exhibited compositions dominated by O, Si, and Al, accompanied by smaller amounts of Mg, Na, K, Ca, Ti, and Cl, depending on the material type. Nanoclay showed the highest Si content (27.49 wt.%), while halloysite and cloisite displayed elemental compositions typical of naturally occurring aluminosilicate minerals. It should be noted that oxygen was intentionally included in the quantitative EDS analysis despite the limitations associated with the detection of light elements. Since oxygen constitutes an essential component of both metal oxides and aluminosilicate structures, its determination was considered necessary to provide a more complete characterization of the investigated nanopowders and to verify the presence of oxide and silicate phases.

### 3.6. Stability of Nano-Additives in Resin

**Figure 6:**
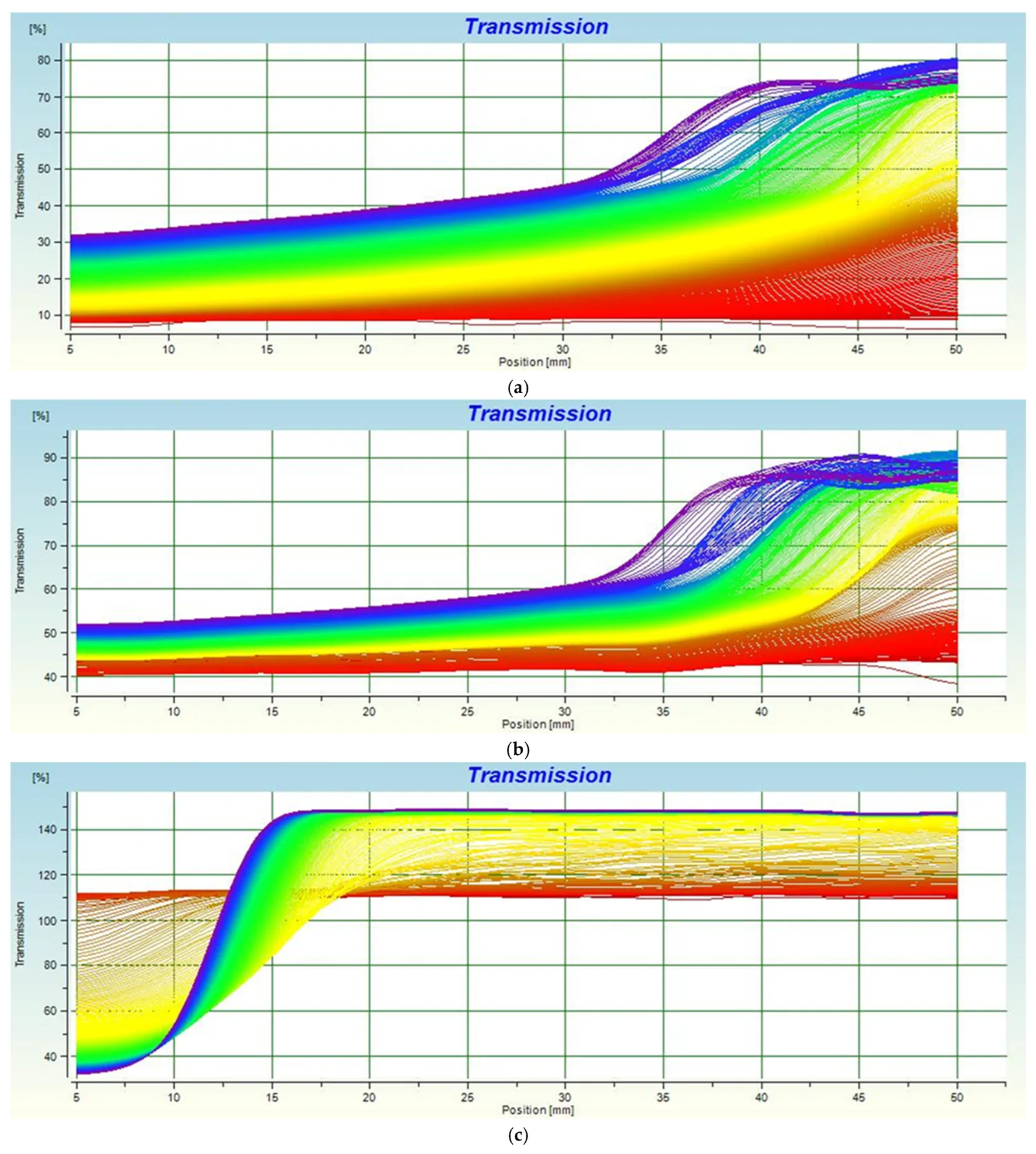

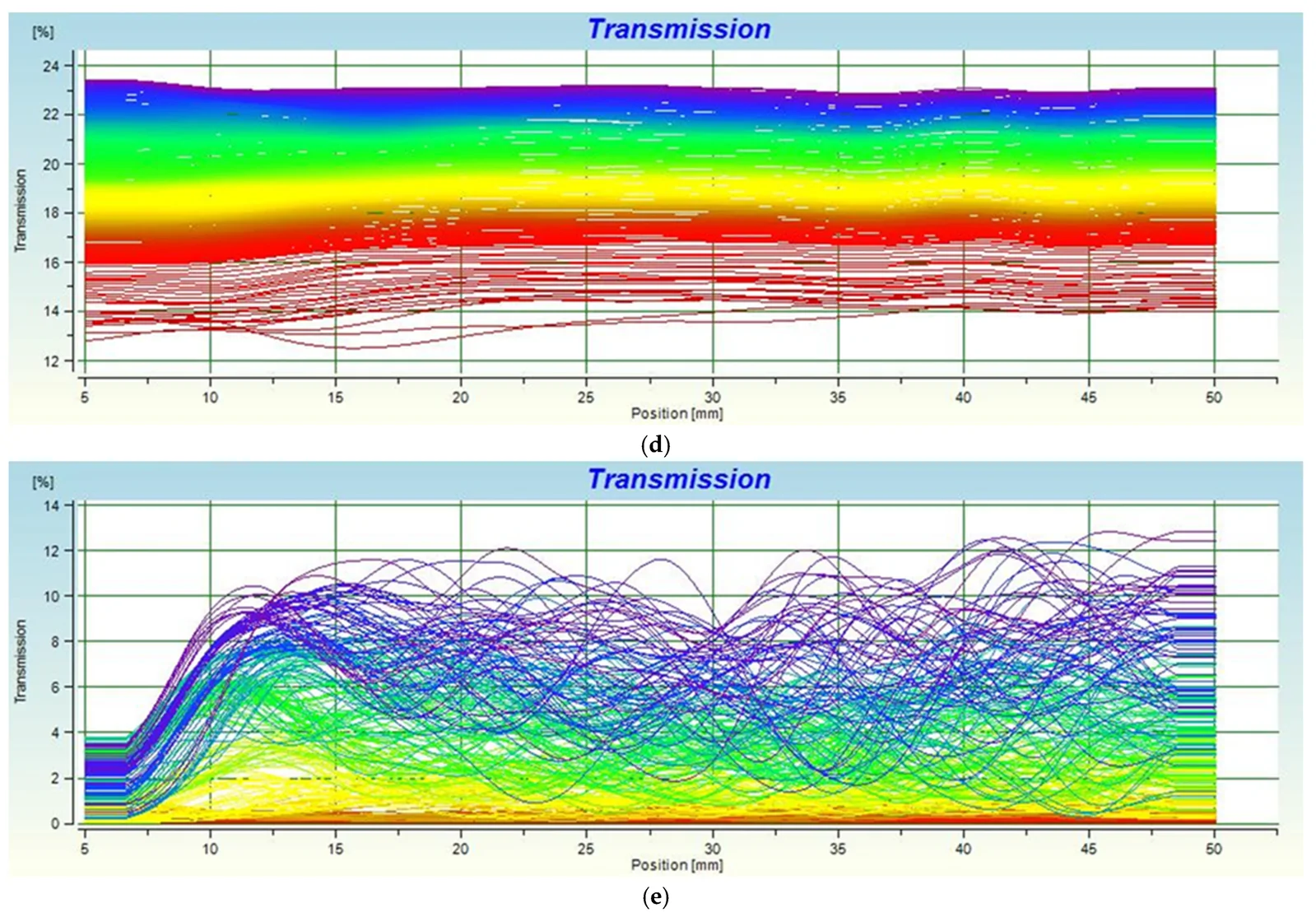
Evolution of transmission profiles recorded during the 20 h stability analysis of unsaturated polyester resin suspensions containing (**a**) Cloisite, (**b**) Halloysite, (**c**) Nanoclay, (**d**) ZnO, and (**e**) CNTs. Changes in transmission along the sample height indicate particle migration, aggregation, and sedimentation processes occurring during storage.

The stability of nanoparticle suspensions in the unsaturated polyester resin was evaluated based on the evolution of transmission profiles recorded over a period of 20 h (Figure 6). Changes in transmission intensity along the sample height provide information on particle migration, sedimentation, and clarification phenomena occurring during storage. In general, an increase in transmission indicates a decrease in the local particle concentration, resulting from particle movement and suspension destabilization. The gravitational settling behavior should also be considered in relation to the properties of the continuous phase and the density of the dispersed materials. The unsaturated polyester resin used in this study exhibits a relatively high dynamic viscosity of 205 mPa·s at 23 °C, which can reduce gravitational settling according to the classical Stokes relationship [57]. The investigated fillers differ considerably in their approximate material densities, with reported values of approximately 1.98 g cm^−3^ for cloisite, 2.53 g cm^−3^ for halloysite, 5.6 g cm^−3^ for ZnO, and approximately 2.1 g cm^−3^ for MWCNTs, as stated in the material safety data sheets. Therefore, differences in density would be expected to contribute to their sedimentation behavior. However, the experimentally observed stability did not follow a simple relationship with filler density. In particular, ZnO, despite having the highest density among the investigated fillers, showed relatively small spatial changes in the transmission profiles and no distinct sedimentation front during the 20 h measurement. This observation indicates that gravitational settling in these systems is additionally controlled by the effective size of dispersed particles and agglomerates, their morphology and agglomeration state, particle-resin interactions, and the relatively high viscosity of the polyester resin.

The halloysite containing suspension exhibited a pronounced increase in transmission in the upper part of the sample. Initially, transmission values ranged from approximately 40%, whereas after 20 h values exceeding around 90% were observed in the upper region of the measurement cell. The formation of a distinct transmission front at approximately 35–45 mm indicates progressive clarification of the resin and sedimentation of halloysite particles. A similar behavior was observed for the cloisite modified resin. The transmission increased to values approaching 80% in the upper part of the sample after 20 h. The gradual displacement of the transmission front confirms continuous particle migration and progressive destabilization of the suspension throughout the measurement period. The nanoclay-containing suspension exhibited a different behavior. During the initial stage of the experiment, the transmission profiles remained relatively stable, suggesting satisfactory short-term dispersion stability. However, significant changes developed at longer measurement times. This behavior suggests delayed destabilization, where initially dispersed particles gradually formed larger agglomerates that subsequently settled under gravity. The ZnO suspension showed the smallest spatial variation of transmission profiles along the sample height. Although the transmission increased from approximately 13% to 23% during the measurement period, the profiles remained nearly parallel, and no distinct sedimentation front was observed. This indicates relatively limited particle migration and a more homogeneous distribution of ZnO particles within the resin matrix compared with the aluminosilicate fillers. The behavior of the CNTs containing suspension differed considerably from that of the mineral nanopowders. The transmission profiles exhibited irregular fluctuations throughout the entire sample height, without the formation of a clearly defined sedimentation front. This behavior is attributed to the anisotropic morphology of carbon nanotubes and their tendency to form entangled networks and agglomerates. Consequently, the destabilization mechanism of CNT suspensions appears to be governed not only by gravitational settling but also by structural rearrangements within the nanotube network.

To complement the qualitative interpretation of the transmission profiles, the migration rate was determined within the 30–40 mm region of the measurement cell. Results are presented in Table 4. The highest migration rate was observed for cloisite (−3.179 ± 0.004 mm h^−1^), followed by halloysite (−1.884 ± 0.009 mm h^−1^), whereas considerably lower values were obtained for nanoclay (−0.373 ± 0.099 mm h^−1^) and ZnO (−0.095 ± 0.001 mm h^−1^). These quantitative results are consistent with the evolution of the transmission profiles, confirming pronounced particle migration for cloisite and halloysite and substantially lower migration for ZnO. The comparatively larger variability observed for nanoclay may be associated with its delayed and less uniform destabilization behavior observed in the transmission profiles. For CNTs, a reliable migration rate could not be determined because no clearly defined migration front was observed and substantial spatial fluctuations occurred throughout the analyzed region.

The present results describe the behavior of nanofillers dispersed in uncured unsaturated polyester resin. Although suspension stability is an important processing parameter, it should not be interpreted as a direct predictor of the reinforcing efficiency or multifunctional performance of the corresponding cured polyester nanocomposites.

## 4. Discussion

The characterization results obtained using XRD, FTIR, SEM/EDS, particle size analysis, and suspension stability measurements provide a comprehensive insight into the physicochemical properties of the investigated nanofillers and their behavior in the unsaturated polyester resin matrix. The weak and poorly resolved FTIR response of CNTs is consistent with the limited infrared activity expected for predominantly graphitic carbon structures. Although EDS indicated an oxygen contribution, the absence of clearly resolved oxygen-containing functional groups in the FTIR spectrum does not allow this oxygen signal to be attributed unambiguously to CNT surface functionalization. The EDS-detected oxygen may originate from surface oxidation or other oxygen-containing species; however, its chemical state cannot be determined by EDS alone. The XRD analysis confirmed the crystalline nature of all investigated nanomaterials and demonstrated good agreement between the experimental diffraction patterns and the corresponding ICDD reference cards. The absence of additional diffraction peaks suggests high phase purity of the analyzed materials. ZnO exhibited sharp and intense diffraction peaks, indicating a highly crystalline structure with a high degree of structural ordering. In contrast, the broader reflections observed for CNTs are characteristic of graphitic carbon materials and indicate a lower degree of long-range crystallographic order. Nevertheless, the dominant (002) reflection confirmed the preservation of the graphitic structure of the nanotubes. The diffraction patterns obtained for Cloisite, halloysite, and nanoclay confirmed the presence of ordered aluminosilicate structures. The characteristic basal reflections observed for these materials indicate the preservation of their layered architecture, which is an important feature influencing their potential interactions with polymer matrices. The similarities observed between the diffraction profiles of these fillers suggest comparable structural characteristics despite differences in their morphology and chemical composition. The differences in crystallinity and structural organization identified by XRD may affect the dispersion behavior of the fillers and their interactions with the polyester resin matrix. The XRD results therefore primarily provide information on the phase identity and structural organization of the investigated nanofillers. Although these structural features are relevant to their overall physicochemical characterization, the present results do not demonstrate a direct effect of crystallinity itself on suspension stability [58]. The observed differences in sedimentation behavior are more appropriately discussed in relation to experimentally observed particle morphology, agglomeration state, and apparent particle size. Consequently, the structural characteristics of the investigated nanomaterials should be considered when assessing their suitability for homogeneous dispersion in polyester resin prior to composite fabrication.

The nitrogen adsorption–desorption analysis revealed substantial differences in the textural properties of the investigated nanofillers. CNTs exhibited by far the highest specific surface area (S_BET_ = 213.728 m^2^/g) and cumulative pore volume (V_cum_ = 0.452 cm^3^/g), which can be associated with the highly developed accessible surface of nanotube assemblies and the void spaces formed within their complex bundled structure. Cloisite also showed a relatively high specific surface area (63.671 m^2^/g) and was the only investigated material for which the t-plot analysis indicated measurable microporosity (S_micro_ = 25.886 m^2^/g and V_micro_ = 0.012 cm^3^/g). In contrast, halloysite, nanoclay, and ZnO exhibited lower specific surface areas and no measurable microporosity according to the t-plot analysis. Particular attention should be given to the CNTs, for which the NLDFT analysis yielded a modal pore width of 0.823 nm, despite the absence of measurable micropore surface area and volume in the t-plot analysis. This apparent discrepancy should be interpreted with caution. Although a carbon-specific NLDFT kernel for N_2_ adsorption at 77 K with slit/cylindrical pore geometry was applied, the calculated pore-size distribution remains model-dependent, and the idealized pore geometry represents a simplification of the complex structure of CNT assemblies. The sub-nanometer mode may therefore reflect adsorption associated with interstitial spaces or grooves between adjacent nanotubes and bundles rather than intrinsic micropores within the nanotubes. Consequently, the DFT-derived value of 0.823 nm should not be regarded as direct evidence of an intrinsically microporous CNT structure. An additional methodological consideration concerns the degassing procedure applied prior to nitrogen adsorption measurements. The same protocol, including a final step at 300 °C, was used for all investigated materials. For organomodified clays, particularly Cloisite 30B, such treatment may partially alter or remove the organic modifier, as thermal degradation of the quaternary ammonium modifier has been reported to begin at substantially lower temperatures [59]. Consequently, the BET surface area and pore-structure parameters obtained for the organomodified clay samples should be interpreted as characteristics of the materials after the applied degassing treatment rather than necessarily representing their unaltered as-received state.

The particle size distributions obtained by laser diffraction represent the equivalent spherical diameters of particles and agglomerates dispersed in water rather than the actual primary dimensions of individual nanostructures. For highly anisotropic materials such as carbon nanotubes, the reported particle size corresponds to the equivalent spherical diameter of nanotube bundles and agglomerates rather than the actual diameter or length of individual nanotubes. Therefore, the particle size values obtained for CNTs should be interpreted as an indicator of their agglomeration state under the applied measurement conditions. The measured D10, D50, and D90 values primarily reflect the dispersion state and agglomeration tendency of the investigated nanomaterials under the applied measurement conditions. It should also be noted that the particle size analysis was performed in distilled water, whereas suspension stability was evaluated in an unsaturated polyester resin. Consequently, the laser diffraction results provide a comparative assessment of the agglomeration state under standardized measurement conditions and should not be interpreted as a direct representation of nanofiller behavior in the resin, where dispersion is additionally influenced by viscosity, polarity, surface energy, and filler–matrix interactions. ZnO exhibited the lowest characteristic particle diameters and the finest dispersion among the analyzed materials. However, the bimodal particle size distribution indicates the coexistence of smaller particles and larger aggregates in the aqueous suspension. In contrast, CNTs showed the highest equivalent D_50_ and D_90_ values, which reflect the dimensions of nanotube bundles and entangled agglomerates, a phenomenon commonly reported for carbon nanotubes due to strong van der Waals interactions [60]. The clay-based fillers (cloisite, halloysite, and nanoclay) exhibited similar particle size distributions and mean particle sizes, suggesting comparable dispersion behavior in water. Slightly higher D_50_ and D_90_ values observed for nanoclay indicate the presence of larger agglomerates compared to cloisite and halloysite. This interpretation is supported by SEM observations, which revealed the presence of larger and more heterogeneous agglomerates in the nanoclay sample compared with the other aluminosilicate fillers. The broad distributions observed for all clay-based materials further confirm the heterogeneous nature of these systems. The observed suspension stability showed only partial agreement with the particle size analysis, indicating that agglomerate size alone does not fully determine sedimentation behavior. Differences between the aqueous and resin media should also be considered when interpreting these results. ZnO, characterized by the lowest median particle diameter (D_50_ = 2.086 µm), exhibited the smallest changes in transmission profiles. In contrast, halloysite and cloisite, with D_50_ values of 8.290 ± 0.041 and 8.996 ± 0.034 µm respectively, showed more pronounced sedimentation phenomena. The observed differences in particle size distribution may influence the filler dispersion within the polymer matrix, affecting the available interfacial area and the efficiency of matrix-filler interactions [61]. Consequently, variations in agglomerate size may influence the structural, mechanical, and functional properties of polyester nanocomposites. Nevertheless, the actual reinforcing effect of the investigated nanofillers should be verified through the characterization of the corresponding cured composite materials. The suspension stability analysis demonstrated that particle size alone cannot fully explain the observed sedimentation behavior. Although ZnO exhibited the smallest particle size and the most uniform morphology, its superior stability was also associated with the absence of large agglomerates and a relatively homogeneous particle distribution. It should be noted that identical sonication conditions do not necessarily produce equivalent dispersion states for nanofillers with different morphologies and agglomeration tendencies. In this study, the same sonication protocol was intentionally applied to all materials to ensure standardized conditions for their comparative evaluation. Therefore, the observed stability reflects nanofiller behavior under common processing conditions rather than individually optimized dispersion. Similarly, the use of an equal mass fraction (0.1 wt.%) does not correspond to equal nanofiller volume fractions or available interfacial areas because of differences in density, specific surface area, and morphology among the investigated materials. Therefore, the present comparison reflects their behavior at a common mass loading rather than under equivalent volumetric or interfacial conditions. The relatively high stability of the ZnO suspension may be attributed not only to its smaller particle size, but also to the more homogeneous morphology and lower degree of agglomeration observed in SEM micrographs. Halloysite and cloisite, despite having similar particle size distributions, showed progressive clarification of the resin and the formation of distinct sedimentation fronts, indicating continuous particle migration during storage. Nanoclay exhibited a different behavior, characterized by relatively stable transmission profiles during the initial stage of the experiment followed by a pronounced increase in transmission at longer times. This observation suggests delayed destabilization, likely resulting from the gradual formation of larger agglomerates that subsequently settled under gravitational forces. Therefore, the stability of nanoclay suspensions appears to be governed not only by particle size but also by interparticle interactions and agglomeration kinetics. The delayed onset of sedimentation suggests that the suspension remained metastable during the initial stage of the experiment, whereas progressive agglomeration occurring over time eventually led to the formation of sedimenting structures. This behavior is consistent with literature reports indicating that particle dimensions, morphology, and agglomeration kinetics can contribute to the stability of such suspensions [62]. The behavior of CNTs differed considerably from that of the mineral nanopowders. Despite exhibiting the largest apparent equivalent agglomerate size determined by laser diffraction, the transmission profiles did not indicate classical sedimentation characterized by a well-defined clarification front. Instead, irregular transmission fluctuations were observed throughout the sample height. This behavior is attributed to the anisotropic morphology of carbon nanotubes and their tendency to form interconnected and entangled networks. This interpretation is consistent with SEM observations, which revealed compact agglomerates and entangled nanotube structures rather than isolated nanotubes. Consequently, the destabilization mechanism of CNTs suspensions appears to be controlled by structural rearrangements and agglomerate formation rather than simple Stokes-type sedimentation [63,64]. Importantly, CNT agglomeration should not be considered solely as an indicator of poor suspension stability, since the final dispersion state and formation of interconnected conductive networks are strongly dependent on processing conditions. Such network formation may be advantageous for applications requiring electrical conductivity, Joule self-heating, or sensing functionality. Recent studies on conductive carbon-based thermosetting nanocomposites have demonstrated that controlling filler dispersion and conductive-network formation can enable efficient self-heating, de-icing, and in situ curing [65]. Therefore, the stability behavior observed here should be regarded as a processing-related characteristic rather than a direct measure of the functional potential of CNTs. The 20 h stability test was performed on uncured resin without the addition of a curing agent and therefore does not directly represent the processing window after cure initiation. The extended observation period was selected to enable comparative evaluation of the long-term tendency of the investigated nanofillers toward sedimentation, agglomeration, and structural rearrangement in the resin. This aspect is particularly relevant when considering nanofiller-modified polyester formulations intended for composite pipe manufacturing, where maintaining a sufficiently homogeneous filler distribution prior to and during processing is important for reproducible material preparation. The quantitative migration-rate analysis was generally consistent with the particle size results, although no simple relationship between particle size and migration rate was observed. ZnO, characterized by the lowest D50 (2.086 ± 0.072 µm), exhibited the lowest absolute migration rate (0.095 ± 0.001 mm h^−1^). In contrast, cloisite and halloysite showed substantially higher migration rates of 3.179 ± 0.004 and 1.884 ± 0.009 mm h^−1^, respectively. Particularly noteworthy is the comparison between the three aluminosilicate fillers. Despite their similar D50 values (8.290–8.996 µm) and D90 values (22.552–24.587 µm), their migration rates differed considerably, with cloisite exhibiting the highest rate, followed by halloysite and nanoclay. This confirms that the sedimentation behavior cannot be explained by apparent particle size alone and is additionally influenced by particle morphology, agglomeration state, interparticle interactions, and interactions with the resin matrix. For CNTs, a reliable migration rate could not be determined because of the absence of a clearly defined migration front and the pronounced spatial fluctuations of the transmission profiles.

Overall, the obtained results indicate that the dispersion stability of nanofillers in unsaturated polyester resin is associated with differences in particle size, morphology, and agglomeration state. Surface-related filler–resin interactions may also contribute to the observed behavior; however, their specific role cannot be established from the present results, as surface energy, wettability, surface charge, and zeta potential were not directly measured. Under the investigated conditions, ZnO exhibited the highest suspension stability, whereas the aluminosilicate fillers and CNTs displayed more complex dispersion phenomena associated with their layered or anisotropic structures. However, higher suspension stability should not be interpreted as superior overall nanofiller performance, since nanofiller selection is application-dependent. Materials exhibiting lower suspension stability, particularly CNTs, may provide specific functionalities associated with the formation of conductive networks. Therefore, the present results primarily describe the dispersion and processing behavior of the investigated nanofillers rather than their overall performance in cured polyester composites. More broadly, the dispersion state of nanofillers can have direct consequences for the functional performance of polymer nanocomposites. Recent studies on stimuli-responsive systems have shown that improved nanoparticle dispersion can promote more homogeneous energy transfer and heating, enabling efficient remotely activated functionalities such as shape-memory actuation and de-icing [66]. These findings highlight the broader technological relevance of controlling nanofiller dispersion and support the importance of physicochemical characterization prior to composite fabrication. Nevertheless, such functional effects were not evaluated in the present study and require direct assessment in the corresponding cured nanocomposites.

It should be emphasized that the present study exclusively focuses on the physicochemical characteristics of the investigated nanofillers and their dispersion stability in uncured unsaturated polyester resin. Although these parameters are important for material processing and nanofiller selection, they should not be interpreted as direct indicators of the reinforcing efficiency or multifunctional performance of the final cured nanocomposites. Such properties additionally depend on curing conditions, filler–matrix interfacial adhesion, dispersion retained after crosslinking, and the resulting composite microstructure.

## 5. Conclusions

The principal value of the present study is the direct comparison of chemically and morphologically distinct nanofillers under a common experimental framework, providing a consistent basis for evaluating their dispersion behavior in unsaturated polyester resin. Their physicochemical properties, morphology, elemental composition, particle size distribution, and dispersion stability were comprehensively characterized in order to evaluate their suitability for polyester-based nanocomposite applications. The combined results highlight the importance of comprehensive characterization of nanofillers prior to their incorporation into polymer matrices. The applied analytical approach enabled the identification of relationships between morphology, particle size, and suspension stability, providing valuable information for the selection and optimization of nanofillers intended for polyester resin modification. The findings presented in this study establish a foundation for selecting nanofillers prior to composite fabrication and provide a basis for future investigations into the influence of nanofiller incorporation on their structure and performance.

## Figures and Tables

**Table 1:** Specific surface area and pore structure parameters of nanomaterials.

Sample	S_BET_ [m^2^/g]	S_micro_ (T-Plot) [m^2^/g]	S_ext_ (T-Plot) [m^2^/g]	S_cum_ (DFT) [m^2^/g]	V_micro_ (T-Plot) [cm^3^/g]	V_cum_ (DFT) [cm^3^/g]	D_mode_ (DFT) [nm]
**Cloisite**	63.671	25.886	38.039	88.508	0.012	0.054	63.671
**Halloysite**	51.828	0	51.828	48.349	0	0.110	12.991
**Nanoclay**	23.345	0	23.345	22.356	0	0.046	23.345
**ZnO**	10.547	0	10.547	10.1143	0	0.012	3.537
**CNTs**	213.728	0	213.728	195.373	0	0.452	0.823

**Table 2:** Characteristic particle size distribution parameters (D_10_, D_50_, D_90_) and mean particle size of the investigated nanopowders. Values are presented as mean ± standard deviation.

Sample Symbol	D_10_ [µm]	D_50_ [µm]	D_90_ [µm]	Mean Size [µm]
**Cloisite**	2.825 ± 0.068	8.357 ± 0.106	22.663 ± 0.063	11.309 ± 0.083
**Halloysite**	2.227 ± 0.016	8.290 ± 0.041	22.552 ± 0.091	11.033 ± 0.042
**Nanoclay**	1.940 ± 0.003	8.996 ± 0.034	24.587 ± 0.073	11.988 ± 0.011
**ZnO**	0.638 ± 0.019	2.086 ± 0.072	11.475 ± 0.724	4.424 ± 0.229
**CNTs**	13.743 ± 0.707	33.511 ± 1.241	55.582 ± 1.902	36.023 ± 1.287

**Table 3:** Elemental composition of the investigated nanopowders determined by EDS analysis, expressed as mass percentage (%M.) and atomic percentage (%At.). “–” indicates that the element was not detected by EDS analysis.

Element	Content [%]	Cloisite	Halloysite	Nanoclay	ZnO	CNTs
O	%M.	47.39 ± 0.24	52.94 ± 0.24	58.81 ± 0.46	17.91 ± 0.15	7.64 ± 0.15
	%At.	51.08 ± 0.26	54.75 ± 0.25	71.90 ± 0.56	30.87 ± 0.26	5.85 ± 0.12
C	%M.	19.32 ± 0.16	21.85 ± 0.15	0.23 ± 0.06	18.40 ± 0.15	92.36 ± 0.16
	%At.	27.74 ± 0.2	30.10 ± 0.21	0.20 ± 0.05	42.26 ± 0.34	94.15 ± 0.16
Zn	%M.	-	-	-	63.69 ± 0.79	-
	%At.	-	-	-	26.87 ± 0.33	-
Si	%M.	19.71 ± 0.12	12.61 ± 0.10	27.49 ± 0.30	-	-
	%At.	12.10 ± 0.08	7.43 ± 0.06	19.14 ± 0.21	-	-
Mg	%M.	2.12 ± 0.04	-	1.54 ± 0.08	-	-
	%At.	1.51 ± 0.03	-	1.24 ± 0.06	-	-
Ti	%M.	-	-	-	-	-
	%At.	-	-	-	-	-
Al	%M.	6.63 ± 0.07	12.59 ± 0.09	8.44 ± 0.16	-	-
	%At.	4.24 ± 0.05	7.72 ± 0.06	6.12 ± 0.12	-	-
Ca	%M.	0.88 ± 0.04	-	0.01 ± 0.05	-	-
	%At.	0.38 ± 0.02	-	0.01 ± 0.03	-	-
Na	%M.	3.94 ± 0.06	-	-	-	-
	%At.	2.95 ± 0.05	-	-	-	-
K	%M.	-	-	-	-	-
	%At.	-	-	-	-	-
Cl	%M.	-	-	1.33 ± 0.08	-	-
	%At.	-	-	0.73 ± 0.04	-	-
Cu	%M.	-	-	2.15 ± 0.29	-	-
	%At.	-	-	0.66 ± 0.09	-	-

**Table 4:** Migration rates of the nanofillers in unsaturated polyester resin.

Sample Symbol	Sedimentation Rate [mm/h]
**Cloisite**	−3.179 ± 0.004
**Halloysite**	−1.884 ± 0.009
**Nanoclay**	−0.373 ± 0.099
**ZnO**	−0.095 ± 0.001
**CNTs**	n.d.

## Data Availability

The original contributions presented in this study are included in the article. Further inquiries can be directed to the corresponding authors.

## Abbreviations

The following abbreviations are used in this manuscript:

CNT/CNTs	Carbon nanotubes
BET	Brunauer–Emmett–Teller
EDS	Energy-dispersive X-ray spectroscopy
FT-IR	Fourier transform infrared spectroscopy
SEM	Scanning electron microscopy
XRD	X-ray diffraction
ZnO	Zinc oxide
